# CircRNF13 Promotes the Malignant Progression of Pancreatic Cancer through Targeting miR-139-5p/IGF1R Axis

**DOI:** 10.1155/2021/6945046

**Published:** 2021-12-03

**Authors:** Xinyuan Liu, Lei Zhou, Yankun Chen, Xueyi Jiang, Jianxin Jiang

**Affiliations:** ^1^School of Clinical Medicine, Guizhou Medical University, Guiyang, Guizhou, China; ^2^Department of Hepatic-Biliary-Pancreatic Surgery, The Affiliated Hospital of Guizhou Medical University, Guiyang, Guizhou, China; ^3^Department of Hepatobiliary Surgery, Renmin Hospital of Wuhan University, Wuhan, China

## Abstract

**Background:**

Mounting evidence has shown circular RNAs (circRNAs) play an important role in the initiation and progression of pancreatic cancer (PC). Meanwhile, circRNAs may serve as the biomarkers for the diagnosis, treatment, and prognosis of PC. Therefore, it is urgent to elucidate the function and underlying mechanism of circRNAs in the development of PC.

**Methods:**

The Cancer-Specific CircRNA Database (CSCD), Circular RNA Interactome database (circinteractome database), and reverse transcription-quantitative polymerase chain reaction (RT-qPCR) were used to verify the expression level of circRNF13 in PC cell lines. Fluorescence in situ hybridization (FISH) and RNase protection assay were used to detect the localization and structure of circRNF13. Then, cell functional experiments were employed to estimate the proliferated, migrated, and invasive abilities in PC. Furthermore, bioinformatic tools, luciferase dual reporter assay, and RT-qPCR were used to investigate the interaction among circRNF13, miR-139-5p, and IGF1R. Eventually, the rescue functional experiments were employed to confirm that circRNF13 targeted the miR-139-5p/IGF1R axis to participate in the development of PC.

**Results:**

CircRNF13 was overexpressed in PC cell lines compared with the normal pancreatic duct cell line. Additionally, inhibition of circRNF13 impaired the proliferation, migration, and invasion of PC cells. CircRNF13 could serve as the molecular sponge of miR-139-5p to inhibit its association with IGF1R that eventually accelerated the malignant progression of PC.

**Conclusion:**

CircRNF13 serves as a competitive endogenous RNA of IGF1R to inhibit the function of miR-139-5p that eventually reinforces the malignant phenotype of PC.

## 1. Introduction

As the seventh leading cause of cancer death, pancreatic cancer (PC) has been viewed as one of the most fetal diseases globally [1]. Growing evidence indicates that age, gender, race, diabetes, family history of PC, genetic factors, chronic infection, non-O blood type, and chronic pancreatitis are main risk factors in PC [2]. The extremely poor prognosis in PC is primarily due to the early onset of metastasis, and therefore, surgical resection is thought to be the mainstay curative treatment for PC patients [3]. Tumor invasion and distant metastasis are the main causes for treatment failure and deterioration of PC. Hence, once the underlying mechanism of PC initialing and progressing is revealed, the curative effect for PC may be enhanced.

CircRNA is a ubiquitously expressed group of noncoding RNAs derived from the back-splicing of pre-mRNAs. Emerging evidence implicates that circRNAs participate in versatile biological processes, such as pluripotency, immune responses, and tumorigenesis. For instance, the elevated expression of circSMARCA5 induced drug sensitivity of breast cancer cell lines *in vitro* and *in vivo* [4]. By detecting the expression level of circRNAs through tissue microarrays, circZKSCAN1 was found to be abnormally expressed in hepatocellular carcinoma (HCC) tissue and involved in the malignant progression of HCC. Additionally, Yang et al. uncovered that circPTK2 was positively correlated with tumor growth and metastasis of colorectal cancer (CRC). Furthermore, their research elucidated that circPTK2 promoted the epithelial-mesenchymal transition (EMT) of colorectal cancer cells by binding to vimentin protein on sites Ser38, Ser55, and Ser82 [5]. Accumulating evidence reveals that numerous miRNA-binding sites existed on diverse circRNAs and mRNA, thus leading to the competition between circRNAs and mRNAs for shared microRNAs (miRNAs), such process is called competing endogenous RNAs (ceRNAs) mechanism. Besides, circRNAs located in the nucleus are involved in transcription regulation. Conn et al. uncovered the nuclear retained CircSEP3 participated in splicing regulation of its linear counterpart. Meanwhile, CircSEP3 bound strongly to its cognate DNA locus and suppressed the binding of the linear RNA at the same sequence [6]. Moreover, circRNAs could be translated or activated through directly binding to protein [7, 8]. Taking together, circRNAs played irreplaceable roles in a variety of biological processes and hence might make a profound impact on medical fields to undermine the relationship between circRNAs and certain diseases.

Previous studies showed that circRNF13 was highly expressed in HBV-associated hepatocellular carcinoma cells. Yan et al. indicated that circRNF13 accelerated the proliferation, migration, and invasion of HBV-associated HCC cells. Mechanistically, circRNF13 served as the sponge of miR-424-5p and inhibited its suppressive function on TGIF2, such process eventually promoted the malignant progression of HBV-associated HCC [9]. However, the role of circRNF13 in PC was rarely studied. For that, we investigated the function of circRNF13 in PC malignant progress and further elucidated its underlying mechanism through *in vitro* and *in vivo* experiments. Our results revealed circRNF13 promoted the proliferation, migration, and invasion of PC cells through sponging to miR-139-5p and upregulated the expression of IGF1R, which eventually led to the accelerated progression of PC. And IGF1R is an effective therapeutic target for cancers. Thus, the circRNF13/miR139-5p/IGF1R axis may pave the way for PC diagnosis and novel therapeutic strategies.

## 2. Materials and Methods

### 2.1. Cell Culture

The normal pancreatic duct epithelial cell line HPDE and different PC cell lines (PANC-1, MIA-PaCa-2, SW1990, BxPC-3, AsPC-1) used in current study were purchased from American Type Culture Collection 124 (ATCC). HPDE, BxPC-3, and AsPC-1 cells were cultured in the Roswell Park Memorial Institute (RPMI) medium (Gibco, USA) supplemented with 10% of decomplemented fetal bovine serum (FBS) (Gibco, USA) and 50 U/mL of penicillin/streptomycin, while others were maintained in high-glucose Dulbecco's modified Eagle medium (DMEM) (Gibco, USA) with FBS and penicillin/streptomycin mentioned above. Cells were washed three times with phosphate-buffered saline (PBS) (Solarbio, Beijing, China) and cultured in a fresh medium before exchange of the medium. Moreover, all the cells were cultured in an appropriate temperature (37°C) and a suitable concentration of carbon dioxide (5% CO_2_).

### 2.2. Cell Transfection

To successfully construct the transfected cells, we planted PAN-1 and MIA-PACA-2 cells uniformly into 6-well plates. When the cultured cells were attached to the bottom of the plate and occupied 60–70% of the well, different reagents were added into different cell lines, respectively. All reagents were synthesized by Ruibo Biotechnology (Guangzhou, China), including circRNF13-si1, circRNF13-si2, miR-139-5p inhibitor, IGF1R overexpressed plasmid, and their corresponding negative controls. Moreover, the Lipofectamine™ 3000 reagent (Invitrogen, USA) was added during the transfection process to improve the transfection efficiency.

### 2.3. RNase Protection Assay

The total RNA extracted from the cell lysate was treated with RNase free water or RNase R (Lucigen) in a reaction buffer at 37°C for 2 h. Consequently, the sample was treated with RNase R inactivation at 65°C for 20 min. Then, the miReasy Mini Kit (QIAGEN) was used to extract the RNA of the indicated sample followed by its elution in water. The data were verified by following RT-qPCR assay.

### 2.4. Cell Counting Kit-8 Assays

The transfected cells were cultured in 96-well plates for 24 h. Then, 10 *μ*L of CCK-8 solution (Boster, China) mixed with 90 *μ*L of medium was added to each well and incubated for 2 h at 37°C. The optional density (OD) at 450 nm was then measured using a microplate reader (BioTek, USA).

### 2.5. EdU Assay

The stably transfected cells were seeded on 96-well plates and cultured in a medium containing 10% fetal bovine serum at 37°C and 5% CO2 for 48 h. EdU assay was then conducted following the manufacturer's protocol (RiboBio, Guangzhou, China) to detect the cell proliferation capability. Images were eventually obtained using a fluorescence microscope (Olympus, Japan).

### 2.6. Wound Healing Assay

While the number of PANC-1 and MIA-PaCa-2 cells stably transfected with corresponding sequence or vector reached 5 *∗* 10^5 in 6-well plates, streaks were created across the monolayer. Cells were then washed twice with PBS and cultured with serum-free DMEM at 37°C and 5% CO_2_ for 24 h to reduce the influence of cell proliferation. Images of the cells were captured at 0 and 24 h after wounding using a microscope (TS100-F; Nikon, Tokyo, Japan).

### 2.7. Transwell Migration and Invasion Assay

For the Transwell migration assay, 5 × 10^4^/ml PANC-1 or MIA-PaCa-2 cells were suspended in the upper chamber of a 24-well Transwell plate and 700 *μ*l of 20% DMEM was placed in the lower chamber. For the Transwell invasion assay, 5  ×  104/ml PANC-1 or MIA-PaCa-2 cells were suspended in the upper chamber of a 24-well Transwell plate pretreated with Matrigel (BD Biosciences) and 700 *μ*l of 20% DMEM was placed in the lower chamber. Cells for migration or invasion assay were counted after 36 h of incubation. Transwell assay images are representative of at least three independent experiments.

### 2.8. RNA Extraction and RT-qPCR Assay

Prior to commencing the study, total RNA was extracted from PANC-1 and MIA-PaCa-2 cells using the TRIzol® reagent (Vazyme Biotech Co., Ltd). The concentration and purity of RNA were then measured using NanoDrop 2000 equipment (Invitrogen, USA). Once the total RNA was extracted, we conducted RT-PCR using the PrimeScript RT Master Mix (Vazyme Biotech Co., Ltd) with corresponding primers to get the complementary DNA (cDNA) of target circRNAs and mRNAs. While the cDNA of target miRNAs was generated using the PrimeScript RT Reagent Kit (Vazyme Biotech Co., Ltd) with specific stem-loop primers. Then, the indicating cDNA was checked by qPCR using the Real-Time PCR Master Mix (SYBR Green; Vazyme Biotech Co., Ltd) in the CFX Connect Fluorescent Quantitative PCR Instrument (Bio-Rad, USA). Different primers were used in our study; U6 was used as the internal reference standard for miR-139-5p, and GAPDH was used as the internal reference standard for circRNF13 and IGF1R. All the primers used are listed below:

 
*β*-actin, forward: GTCTGCCTTGGTAGTGGATAATG and reverse: TCGAGGACGCCCTATCATGG
  IGF1R, forward: TCGACATCCGCAACGACTATC and reverse: CCAGGGCGTAGTTGTAGAAGAG
  CircRNF13, forward: CCTTATCATAGTGGGCATCTGTC and reverse: AGCATCTCGTTGTAAAATCACCTT
  miR-139-5p, forward: ACACTCCAGCTGGGGACCTCTGTGCACGTG and reverse: TGGTGTCGTGGAGTCG
  U6, forward: CTCGCTTCGGCAGCACA and reverse: AACGCTTCACGAATTTGCGT

### 2.9. Western Blotting

Total proteins were extracted using the RIPA lysis buffer and quantified by bicinchoninic acid (BCA, Boster, China). The extracted proteins were then loaded and separated on 10% SDS-PAGE. Then, the isolated proteins were transferred to polyvinylidene fluoride (PVDF) membranes (Boster, China). After 2 hours of blocking, the corresponding primary antibodies were used to incubate target proteins maintained on the PVDF membranes. The primary antibodies (Abcam, USA) used in our study were anti-IGF1R (1 : 500), anti-*β*-actin (1 : 1000), anti-CDK4 (1 : 500), anti-CDK6 (1 : 500), and anti-Cyclin D1 (1 : 500). Following incubation, the membranes were soaked in goat anti-rabbit secondary antibody (Abcam, USA) at room temperature for 2 h. Eventually, the processed membranes were dripped with the ECL chemiluminescent 199 reagent (Servicebio, Wuhan, China) and scanned using the ChemiDoc XRS+ system (Bio-Rad).

### 2.10. Fluorescence In Situ Hybridization (FISH)

The FISH probe for circRNF13 labeled with 6-FAM was utilized in our study. After the cultured cells were attached to slides, they were treated with 4% paraformaldehyde for immobilizing and washed with PBS. Consequently, the protease K (Sangon, Shanghai, China) was used to digest processed cells. After PBS washing, the cells were treated by 1% paraformaldehyde followed by alcohol dehydration. Eventually, the hybridization solution dilute probe was added onto the cell slides and denatured at 73°C for 3 min, then hybridized overnight at 42°C, and washed with preheated 50% formamide/2 × SSC, 0.1% NP40/2 × SSC, and DAPI staining solution at room temperature. Images were obtained using a laser confocal microscope (Leica, Mannheim, Germany).

### 2.11. Bioinformatic Analysis

Targets for circRNF13 were predicted using the R software package combining CSCD and circinteractome databases. Meanwhile, the possible functions of circRNF13 in PC were analyzed through Gene Ontology (GO) functional annotation and Kyoto Encyclopedia of Genes and Genomes (KEGG) pathway analysis. Furthermore, the underlying interaction among circRNF13, possible miRNA, and its target gene was displayed in the form of Sankey diagram through protein-protein interaction (PPI) analysis.

### 2.12. Luciferase Reporter Assay

Following sequence comparisons of circRNF13, IGF1R, and miR-139-5p, fluorescein-labeled reporter gene detection was performed using a dual luciferase assay system kit (Promega) according to the manufacturer's instructions. Wild-type and mutant circRNF13 or IGF1R 3'UTR dual luciferase reporter vectors incorporating miRNA-binding sites were purchased from Guangzhou Yingxin Co, Ltd. (Guangzhou, China). PANC-1 and MIA-PaCa-2 cells were cotransfected with mentioned wild-type or mutant vectors using Lipofectamine™ 3000 reagent (Invitrogen). After 48-h incubation, each well received passive lysis buffer, and the supernatant was collected for detection. The absorbance of each well was measured at 580 nm using a microplate reader after adding the passive lysis buffer sample and luciferase assay reagent II. Eventually, Stop & Glo^®^ Reagent was added to the same well, and the absorbance was measured at 460 nm.

### 2.13. Construction of PC-Bearing Nude Mouse Model and Immunohistochemistry (IHC) Experiment

To identify the effect of circRNF13 on PC cells *in vivo*, we managed to construct a PC-bearing nude mouse model with intratumoral injection of circRNF13 siRNA. The tumor size and weight were measured, and IHC was performed as previously described [10]. Tissues sections were incubated with antibodies against PCNA (CST) or ki67 (CST) at 4°C overnight. ChemMate DAKO EnVision Detection Kit (Dako) was used for immunostaining.

### 2.14. Statistical Analysis

All data were expressed as mean ± standard deviation. SPSS 21.0 statistical software and GraphPad Prism 8.0 were used to analyze data. Statistical significance was analyzed using Student's *t* test and one-way analysis of variance (ANOVA). Statistical significance was considered at a *P* value less than 0.05.

## 3. Results

### 3.1. CircRNF13 Is a Typical Circular RNA and Was Overexpressed in PC Cells

**Figure 1:**
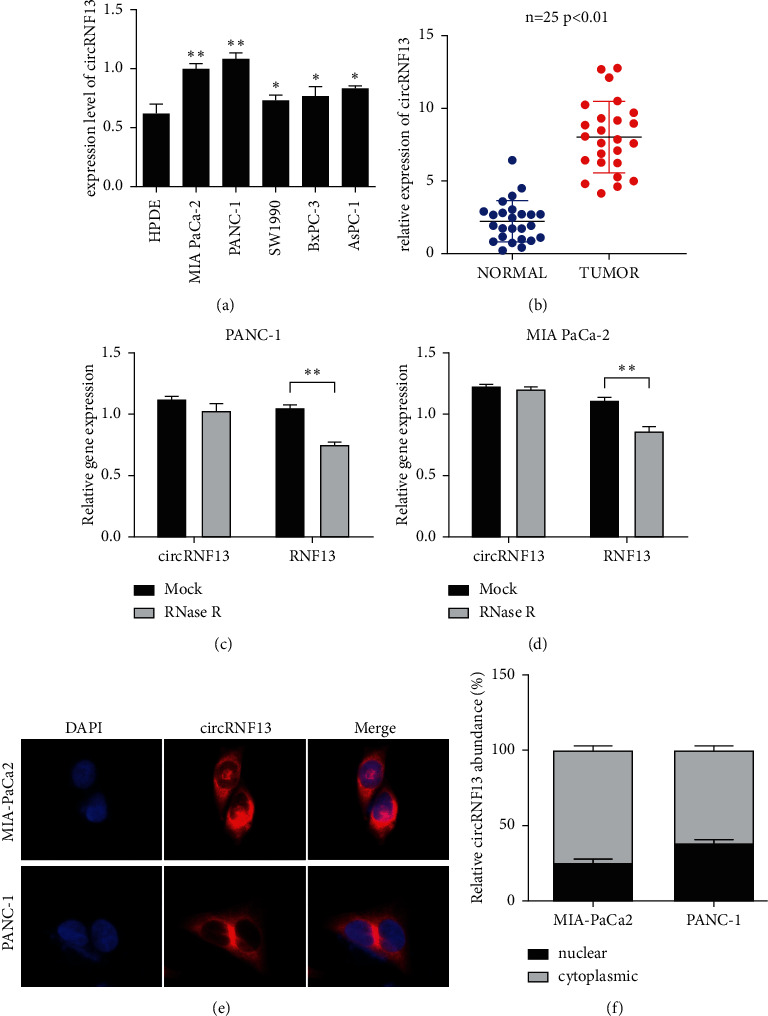
CircRNF13 is overexpressed in pancreatic cancer. (a) CircRNF13 expression in PC cell lines was tested by RT-qPCR. (b) CircRNF13 expression in pancreatic cancer tissues were tested by RT-qPCR. (c, d) RNase protection assay was conducted to explore the structure of circRNF13 in PANC-1 and MIA-PaCa-2 cell lines. *∗∗* indicates *P* < 0.01. (e, f) FISH assay was performed to confirm the localization of circRNF13 in PANC-1 and MIA-PaCa-2 cells.

To investigate the expression level of circRNF13 in PC cell lines, we conducted the RT-qPCR experiment, and the result showed that circRNF13 expression was upregulated in PC cell lines compared with the normal pancreatic duct cell line (HPDE) (Figure 1(a)). Meanwhile, circRNF13 was most highly expressed in PANC-1 and MIA-PaCa-2 cell lines. Moreover, RT-qPCR analysis indicated that circRNF13 expression was upregulated in 25 pairs of PC tissues (Figure 1(b)). Furthermore, the stability of circRNF13 in PC cells treated with RNase R was verified through the RT-qPCR experiment, and the result revealed that circRNF13 had a stronger resistance to RNase R treatment compared with the linear counterpart of RNF13 (Figures 1(c) and 1(d)). To explore the subcellular distribution of circRNF13, we performed the FISH assay and proved that circRNF13 was predominantly localized at the cytoplasm of PC cell (Figures 1(e) and 1(f)). All results mentioned above indicated that circRNF13 may be involved in the tumorigenesis of PC. Meanwhile, as circRNF13 was predominantly localized at the cytoplasm of PC cell, it probably functioned as the molecular sponge of its target miRNA during PC progression.

### 3.2. CircRNF13 Accelerated the Proliferation, Migration, and Invasion of PC Cells

**Figure 2:**
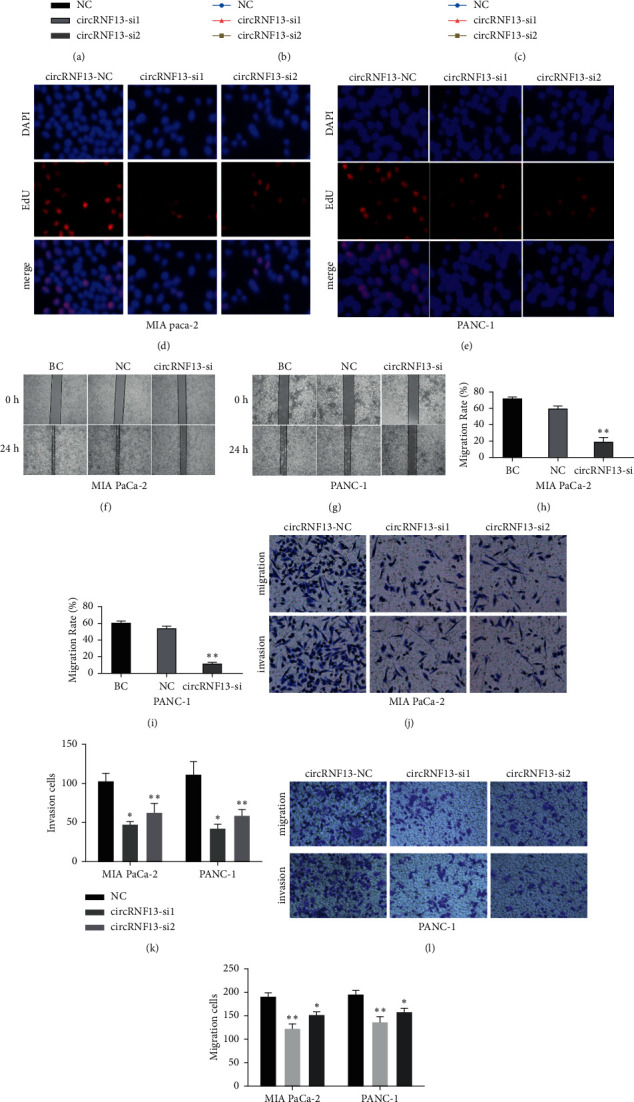
CircRNF13 knockdown inhibits pancreatic cancer cell proliferation and invasion. (a) The expression of circRNF13 in circRNF13-silenced PC cells. (b, c) CCK-8 assay was conducted on MIA-PaCa-2 or PANC-1 cell lines transfected with circRNF13 siRNA. *∗∗* indicates *P* < 0.01. (d, e) EdU assay was performed on MIA-PaCa-2 or PANC-1 cell lines. (f–i) Wound healing experiment displayed decreased invasive capability of MIA-PaCa-2 or PANC-1 cell lines after transfection of circRNF13-si1. (h, i) The quantification of wound healing experiment was shown in the form of bar plot. (j, l) Transwell migration (j) and invasion (l) assays were applied to evaluate the invasiveness and migration capabilities of circRNF13-silenced PC cells. (k, m) The quantification of Transwell migration (k) and invasion (m) assay was shown in the form of bar plot. *∗* indicates *P* < 0.05, *∗∗* indicates *P* < 0.01.

Based on previous studies, circRNF13 was involved in the proliferation and metastasis of lung adenocarcinoma [11], and we speculated that circRNF13 might be a regulator of PC cell proliferation and metastasis. Herein, we conducted CCK-8 assay, EdU assay, Transwell assay, and wound healing assay to illustrate the function of circRNF13 in PC cells. CCK-8 assay suggested that after successfully being transfected with circRNF13 silencing sequence (siRNA) (Figure 2(a)), the cell viability decreased rapidly in PANC-1 and MIA-PaCa-2 cell lines (Figures 2(b) and 2(c)). In addition, EdU assay confirmed a significant inhibition on cell growth of PC cells pretreated with circRNF13 siRNA (Figures 2(d) and 2(e)). The wound healing assay showed that the migration ability of PC cells transfected with circRNF3 siRNA was attenuated compared with the negative control group (Figures 2(f) and 2(g)). Furthermore, we used Transwell assay to measure the cell migration and invasion, and the results illustrated that the downregulation of circRNF13 expression led to a dramatic decrease in the migration and invasion capability of PC cells (Figures 2(j)–2(m)). Taken together, our experiments elucidated that circRNF13 accelerated the proliferation, migration, and invasion of PC cells.

### 3.3. MiR-139-5p/IGF1R Axis Is Involved in circRNF13-Mediated PC Malignancy

**Figure 3:**
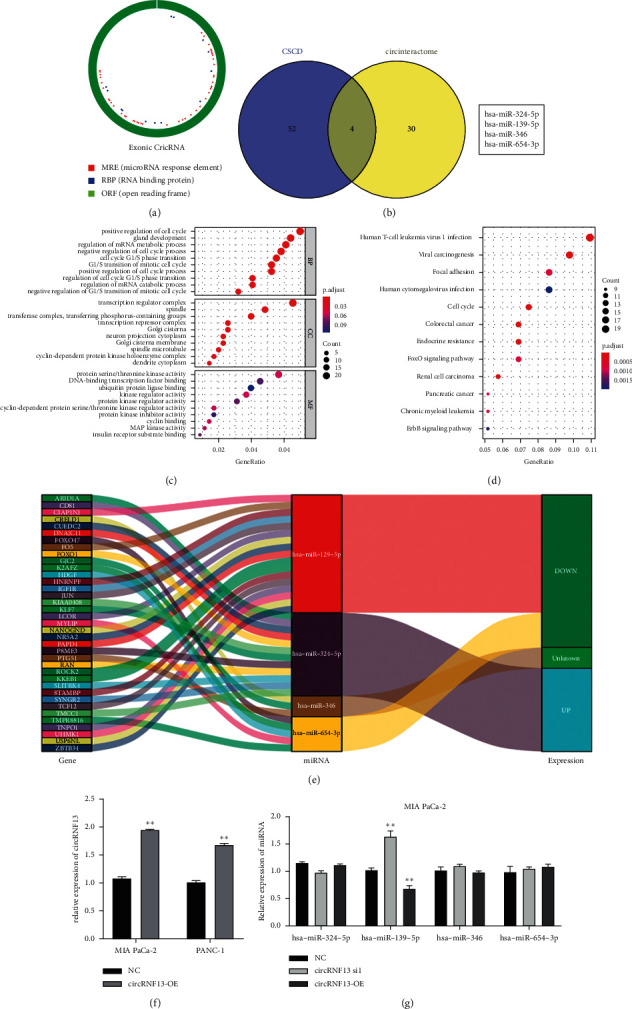
Bioinformatic predication of the possible target of circRNF13 and the relative biological process. (a) The sequence and the binding loci in circRNF13 were showed to investigate the possible target of circRNF13. (b) The possible target miRNAs were analyzed combining CSCD and circinteractome databases, and the result was shown in the form of Venn diagram. (c, d) The GO (c) and KEGG (d) analyses were conducted to explore the relative biological process involving circRNF13. (e) Sankey diagram showed the predicted targets associated with circRNF13 and its possible target miRNA. (f) The expression of circRNF13 in PC cells transfected with circRNF13 overexpression plasmid. (g) The expression of potential target genes of circRNF13 in PC cells transfected with circRNF13 siRNA. *∗∗* indicates *P* < 0.01.

To explore the deep molecular mechanisms by which circRNF13 mediated the malignant phenotypes of PC, we utilized bioinformatics databases including circinteractome (https://circinteractome.irp.nia.nih.gov/) and CSCD databases (http://gb.whu.edu.cn/CSCD/) to screen the potential targets of circRNF13. Subsequently, miR-324-5p, miR-139-5p, miR-346, and miR-654-3p were predicted to be the potential target of circRNF13 (Figures 3(a) and 3(b)). Meanwhile, GO and KEGG enrichment analysis showed potential biological function and pathways that circRNF13 might be involved (Figures 3(c) and 3(d)). To further precisely verify the exact target of circRNF13, we constructed the circRNF13 overexpression vector in PANC-1 and MIA-PaCa-2 cells and analyzed the expression of four candidate target genes by RT-qPCR. The results showed that the expression of miR-139-5p was significantly reduced in PC cell lines transfected with circRNF13-overexpressed plasmid (Figures 3(f) and 3(g)). Accordingly, we speculated that circRNF13 might regulate the malignant progression of PC through targeting miR-139-5p. Moreover, we found that MAP kinase activity, cyclin-dependent protein serine/threonine kinase regulator activity, and insulin receptor substrate binding were downstream processes of circRNF13 in GO and KEGG analysis (Figures 3(c) and 3(d)). Therefore, we speculated the typical biomarkers, such as IGF1R CDK4, CDK6, and cyclinD1, in these pathways that might be involved in the circRNF13-mediated PC malignant progression. It is well known that IGF1R contributes to the abnormal biological characteristics of PC, we hypothesized that IGF1R may be a downstream target of circRNF13.

### 3.4. CircRNF13 Accelerates PC Cancer Progression through Targeting miR-139-5p/IGF1R Axis

**Figure 4:**
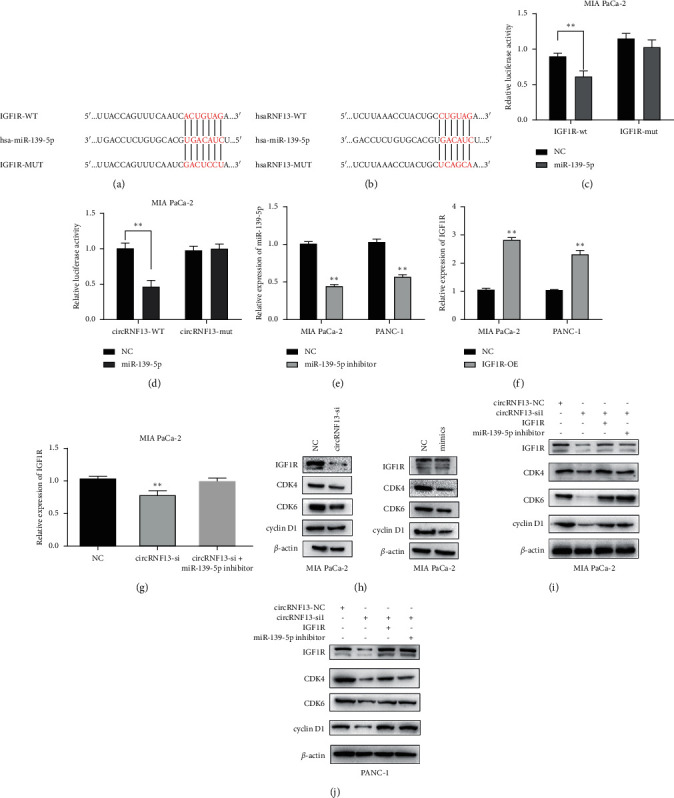
The interaction among circRNF13, miR-139-5p, and IGF1R. (a–d) Dual luciferase reporter assay was conducted to verify the combination of circRNF13 with miR-139-5p and miR-139-5p with IGF1R. *∗∗* indicates *P* < 0.01. The sequence of genes mentioned above and corresponding mutant sequence were shown in (a) and (b). (e) The expression of miR-139-5p in PC cells transfected with miR-139-5p. (f) The expression of IGF1R in PC cell lines transfected with IGF1R overexpressed plasmid. (g) RT-qPCR showed the relative expression of IGF1R in PC cells after transfection of circRNF13 siRNA or cotransfection of circRNF13 and miR-139-5p inhibitor. *∗∗* indicates *P* < 0.01. (h) Western blot experiment indicated the relative expression of IGF1R, CDK4, CDK6, or cyclinD1 in MIA-PaCa-2 cell transfected with circRNF13 siRNA or miR-139-5p mimics, respectively. Western blot experiment showed the association among circRNF13, miR-139-5p, and IGF1R. (i, j) Alteration of IGF1R CDK4, CDK6, and cyclinD1 expression in MIA-PaCa-2 (i) or PANC-1 (j) after different treatments were analyzed by the western blot experiment.

To elucidate the relationship among miR-139-5p, IGF1R, and circRNF13 in PC progression, we conducted the luciferase reporter assay on MIA-PaCa-2 cell that successfully transfected with the mutant of IGF1R or circRNF13, respectively (Figures 4(a) and 4(b)). Our results demonstrated that miR-139-5p could bind to IGF1R or circRNF13, respectively (Figures 4(c) and 4(d)). Furthermore, RT-qPCR verified that the expression of miR-139-5p was negatively correlated with IGF1R (Figures 4(e) and 4(f)). And western blot experiments in MIA-PaCa-2 cells demonstrated that the expression of IGF1R, CDK4, CDK6, or cyclinD1 was decreased in the cricRNF13-knockdown group. Moreover, the expression of IGF1R was decreased after the transfection of miR-139-5p mimics (Figure 4(h)). The above results indicated that circRNF13 was positively correlated with IGF1R expression, and miR-139-5p was negatively correlated with IGF1R expression. Next, we designed the corresponding rescue experiment, that is, to analyze the expression of IGF1R in PC cells cotransfected with circNF13 siRNA and miR-139-5p inhibitor. Meanwhile, the expressions of IGF1R, CDK4, CDK6, and cyclinD1 were quantified by western blot experiments after circRNF13-siRNA and IGF1R overexpression plasmids were transfected simultaneously (Figures 4(i) and 4(j)). The results showed that compared with the circRNF13-siRNA group, the IGF1R, CDK4, CDK6, and cyclinD1 expressions were rebounded in the cotransfection group.

### 3.5. MiR-139-5p/IGF1R Axis Was Exactly the Target of circRNF13 to Regulate the Development of PC

**Figure 5:**
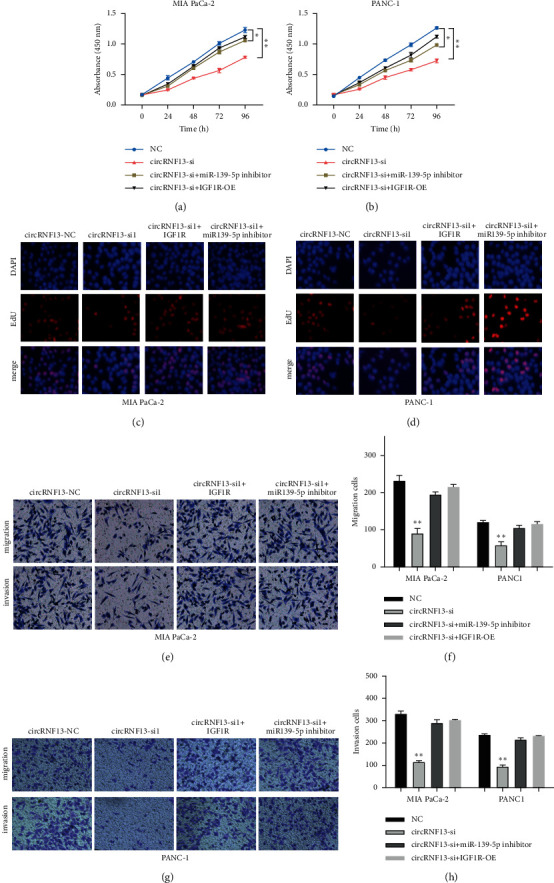
CircRNF13 regulates the progression of pancreatic cancer through targeting miR-139-5p/IGF1R axis. (a, b) CCK-8 assay implicated the growth ability of MIA-PaCa-2 (a) or PANC-1 (b) cell lines on the circRNF13 silence group or cotransfection group. “*∗∗*” indicates *P* < 0.01. (c, d) EdU assay showed the effect of circRNF13 siRNA on proliferation of MIA-PaCa-2 (c) or PANC-1 (d) cell lines after the cotransfection of miR-139-5p inhibitor or IGF1R overexpressed plasmid with circRNF13 siRNA. (e, g) Transwell assay displayed the invasion and migration ability of MIA-PaCa-2 (e) or PANC-1 (g) cell on cotransfection group. (f, h) The quantification of Transwell migration (f) and invasion (h) assay. *∗∗* indicates *P* < 0.01.

To verify that circRNF13 regulates malignant progression of PC through the miR-139-5p/IGF1R axis, we performed CCK-8 assay, EDU assay, wound healing assay, and Transwell assay. CCK-8 assay showed that compared with PC cells transfected with circRNF13 siRNA, the PC cell group cotransfected with circRNF13 siRNA and miR-139-5p inhibitor recovered cell viability (Figures 5(a) and 5(b)). EdU assay showed that the proliferation ability of the cotransfection group rebounded compared with the circRNF13-siRNA-transfected group (Figures 5(c) and 5(d)). Wound healing assay and Transwell assay showed that the invasion and migration ability of the cotransfection group recovered and was similar to that of the negative control group. Similar results were observed in PC cell lines cotransfected with circRNF13-siRNA-overexpressing and IGF1R-overexpressing plasmids (Figures 5(e)-5(g)). In summary, our results suggested that circRNF13 promoted PC proliferation, invasion, and migration by targeting the miR-139-5p/IGF1R axis.

### 3.6. Silencing of circRNF13 Impaired the Proliferation of PC *In Vivo*

**Figure 6:**
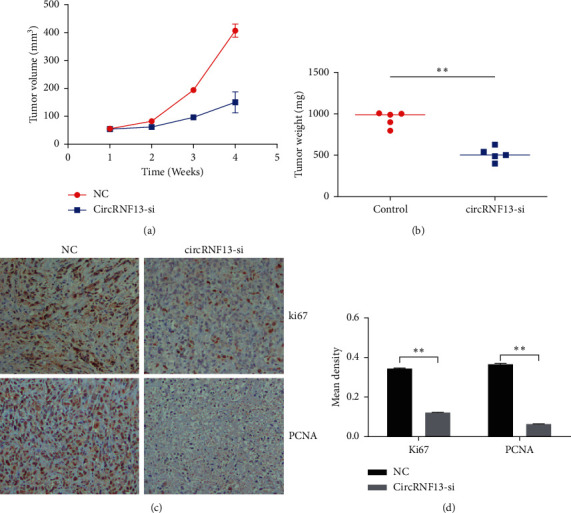
CircRNF13 accelerates the proliferation of pancreatic cancer *in vivo*. (a) The quantification of tumor volume in PC-bearing mice. (b) The quantification of tumor weight in PC-bearing mice. *∗∗* indicates *P* < 0.01. (c) IHC was conducted to confirm the effect of circRNF13 on tumor growth using ki67 or PCNA as a biomarker of pancreatic cancer proliferation. (d) The quantification of IHC results. *∗∗* indicates *P* < 0.01.

Through establishing the PC-bearing nude mice, we compared the proliferation capability between PC cells with or without circRNF13-siRNA injection. As our data showed, after the intratumoral injection of cholesterol-conjugated circRNF13 siRNA to PC-bearing nude mice, the proliferation of PC decreased rapidly (Figures 6(a) and 6(b)). Moreover, the expressions of ki67 and PCNA were markedly reduced in the tumor tissue of PC-bearing nude mice (Figures 6(c) and 6(d)). Taking together, our experiment verified that the silencing of circRNF13 attenuated the proliferation of PC *in vivo*.

## 4. Discussion

PC is among the top 3 leading causes of cancer death worldwide, and its motility and mobility remained high though considering its promotion in diagnosis and therapy. Nonetheless, PC used to be a rare disease and remains rare in less developed countries [11]. The occurrence of PC is closely related to age, and the incidence of PC has increased in aging population [12]. PC is characterized with poor prognosis [13], and the diagnosis of PC remains one of the greatest medical challenges [14, 15]. Therefore, it is urgent for researchers to find proper diagnostic and therapeutic methods for PC.

CircRNA is a newly discovered type of noncoding RNA with a toroidal structure in the length over 200 bp and resisted degradation by RNase [16]. As a particular class of long noncoding RNAs (lncRNAs), circRNAs are involved in a variety of physiological and pathological processes, including cardiovascular disease initialing [17], immune response, stress response, and tumorigenesis [18–20]. CircRNAs usually function as the molecular sponge of miRNA and inhibits the efficacy of miRNA on the target gene [21, 22]. Previous studies have identified mounting types of circRNAs playing irreplaceable roles in carcinogenesis [23–25]. Wang et al.' study revealed that circRNA_002178 could act as a ceRNA to promote PDL1/PD1 expression in lung adenocarcinoma [26]. Liu et al. found circRNA_100367 regulated the radiation sensitivity of esophageal squamous cell carcinomas through the miR-217/Wnt3 pathway [27]. Huang's research showed hsa_circRNA_104348 promotes hepatocellular carcinoma progression through modulating miR-187-3p/RTKN2 axis and activating Wnt/*β*-catenin pathway [28]. Moreover, in PC, circNFIB1 was found to inhibit lymphangiogenesis and lymphatic metastasis via the miR-486-5p/PIK3R1/VEGF-C axis [29]. Huang's study demonstrated that circRNA_000864 upregulated B-cell translocation gene 2 expression and repressed migration and invasion in PC cells by binding to miR-361-3p [30].

CircRNF13 was found to regulate the invasion and metastasis in lung adenocarcinoma by targeting miR-93-5p [31]. Zhang's research found circRNF13 inhibited the proliferation, migration, and invasion of acute myeloid leukemia by regulating the expression of miRNA-1224-5p [32]. However, little was known about circRNF13 in PC. In this study, we discovered for the first time that circRNF13 promoted the proliferation, migration, and invasion of PC. Functional experiments showed that knockdown of circRNF13 significantly inhibited the invasion and migration of PC cells. To explore the underlying mechanism, we first investigated the subcellular location. The results demonstrated that circRNF13 was mainly located in the cytoplasm using FISH assay. Accordingly, we inferred that circRNF13 might serve as a molecular sponge to bind miRNAs and affect the expression of its downstream target genes, which ultimately leads to phenotypic changes in PC. Through bioinformatics analysis, we screened out four candidate target genes that were highly correlated with circRNF13, including miR-324-5p, miR-139-5p, miR-346, and miR-654-3p. Furthermore, RT-qPCR experiments determined that miR-139-5p was the potential target of circRNF13. MiR-139-5p has been found to significantly reduce the migration and proliferation of anaplastic thyroid cancer cells [33], while in our study we elucidated the role miR-139-5p played in PC for the first time. Subsequently, combining our conjecture with GO analysis and KEGG analysis, we screened IGF1R as the target gene of miR-139-5p. The dual luciferase reporter gene experiments confirmed our conjecture. Previous studies revealed that IGF-1R was critically involved in PC pathophysiology, promoting cancer cell survival and therapeutic resistance [34, 35]. Eventually, rescue experiments further confirmed that circRNF13 ultimately promoted the malignant phenotype of PC by targeting the miR139-5p/IGF1R axis.

## 5. Conclusion

CircRNF13 promotes the proliferation, invasion, and migration of PC, which provides the possibility to discover new molecular markers of PC. The circRNF13/miR139-5p/IGF1R signal axis plays key roles in the malignant progression of PC. Regulating the function of this signal axis may improve the diagnosis and treatment of PC in the future.

## Figures and Tables

**Figure 7:**
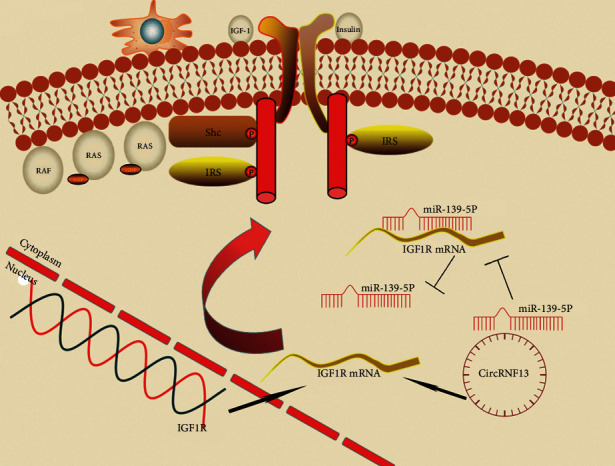
Schematic diagram of this study. Circular RNF13 promotes the malignant progression of pancreatic cancer through targeting the miR-139-5p/IGF1R axis.

## Data Availability

All data generated and analyzed during this study are included in this published article and are available on request.

## Abbreviations

circRNAs: Circular RNAs
CSCD: Cancer-Specific CircRNA database
RT-qPCR: Reverse transcription-quantitative polymerase chain reaction
FISH: Fluorescence in situ hybridization
CCK-8: Cell Counting kit-8 assay
HCC: Hepatocellular carcinoma
CRC: Colorectal cancer
ceRNAs: Competing endogenous RNAs
ATCC: American Type Culture Collection 124
FBS: Fetal bovine serum
RPMI: Roswell park memorial institute
DMEM: Dulbecco's modified Eagle medium
PBS: Phosphate-buffered saline
BCA: Bicinchoninic acid
PVDF: Polyvinylidene fluoride
GO: Gene Ontology
KEGG: Kyoto Encyclopedia of Genes and Genomes
PPI: Protein-protein interaction
siRNA: Silencing sequence
lncRNAs: Long noncoding RNA
miRNAs: MicroRNAs.

## Supplementary Materials

Supplementary Materials Supplemental Figure 1: the raw data of western blot showed in Figure 4H and Figure 4I. (a)–(j) represent the corresponding western blot results in Figure 4H, and K-O represent western blot results in Figure 4I. Supplemental Figure 2: the raw data of western blot showed in Figure 4J. (a)–(e) represent the corresponding western blot results in Figure 4J.
