# Supplementary figures and images for: CircRNF13 Promotes the Malignant Progression of Pancreatic Cancer through Targeting miR-139-5p/IGF1R Axis
Source: J Oncol. 2021 Dec 3;2021:6945046. doi: 10.1155/2021/6945046 (PMC8664507; doi:10.1155/2021/6945046)

Supplemental Figure1

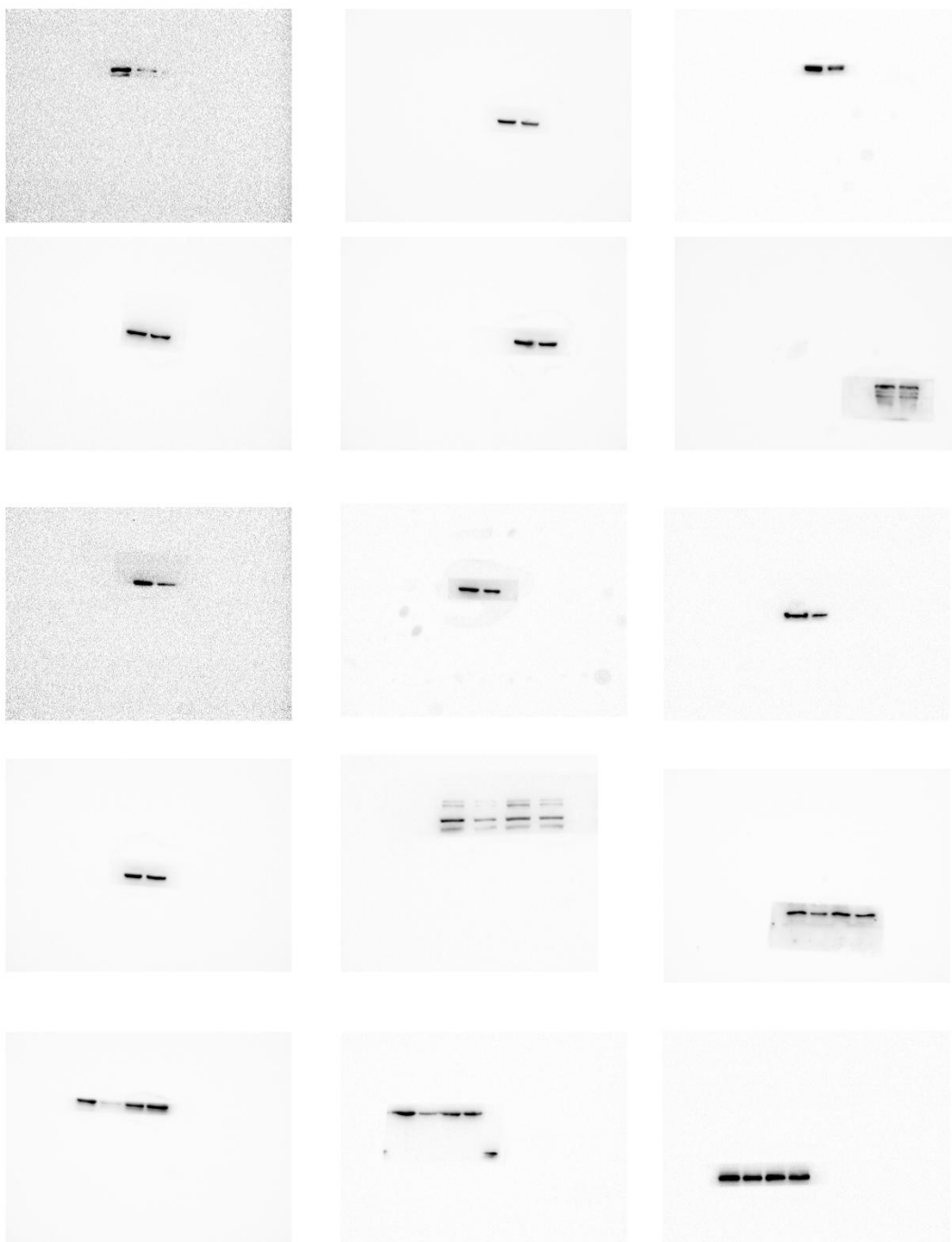

Supplemental Figure2

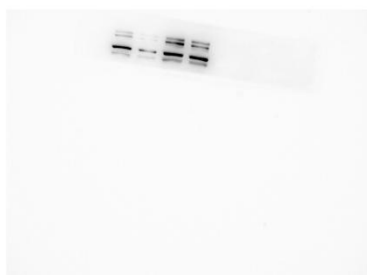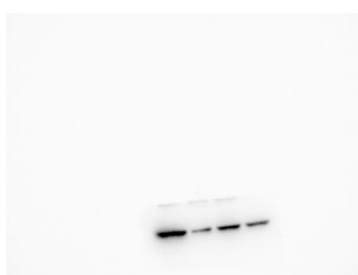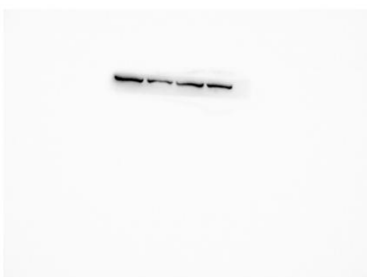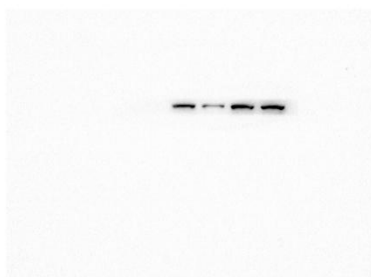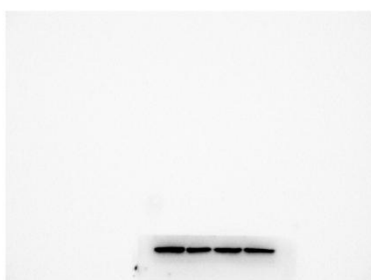

Supplement: Supplementary Materials — Supplemental Figure 1: the raw data of western blot showed in Figure 4H and Figure 4I. (a)–(j) represent the corresponding western blot results in Figure 4H, and K-O represent western blot results in Figure 4I. Supplemental Figure 2: the raw data of western blot showed in Figure 4J. (a)–(e) represent the corresponding western blot results in Figure 4J. [file 6945046.f1.pdf]
